# 2175. Diagnostic Utility of Pneumococcal Urinary Antigen Assay in Clinical Practice: a Retrospective Study in a Community Hospital in Evanston, Illinois

**DOI:** 10.1093/ofid/ofac492.1795

**Published:** 2022-12-15

**Authors:** Goar Egoryan, Guillermo Rodriguez Nava, Guillermo Rodriguez Nava, Armen Kishmiryan

**Affiliations:** Ascension Health Saint Francis Hospital, Evanston, Illinois; Stanford University School of Medicine, Palo Alto, California; Stanford University School of Medicine, Palo Alto, California; Ascension Health, Evanston, Illinois

## Abstract

**Background:**

While the pneumococcal urine antigen test (PUAT) is a validated tool for diagnosing pneumonia, recent evidence suggested lower sensitivity than expected (approximately 60-65%) and limited impact on clinical outcomes. A lack of clinical indicators for a positive test result and the absence of clear recommendations based on high-quality evidence create grounds for potential test misuse in real practice.

**Methods:**

We performed a Workbench report to retrieve individual orders of *Streptococcus pneumoniae* urine antigen test (BinaxNOW^TM^) from our community hospital in Evanston, Illinois, between January 1, 2011, and December 31, 2021. We then identified patients with or without clinical pneumonia based on McGeer criteria: new infiltrates on chest x-ray plus cough, shortness of breath, oxygen saturation < 94%, or respiratory rate ≥ 25 plus fever, leukocytosis, or altered mental status.

**Results:**

Among 8,110 individuals tested for PUAT, only 273 positive results were identified (3.36%); of those, 83 (30.4%) did not meet criteria for clinical pneumonia **(Figure 1)**. The median age of patients in a group with clinical pneumonia and without was 67 and 69 years, respectively. The prevalence of diabetes mellitus, COPD/asthma, and immunosuppression was not statistically significant between groups **(Table 1)**. The signs and symptoms are presented in **Table 2.***S. pneumoniae* was cultured in only 13.9% of cases in patients with clinical pneumonia, which correlates with the known sensitivities of this test **(Table 3)**. More ICU admissions and NIPPV use were noted in patients with clinical pneumonia (p = 0.04 each). However, there was no difference in the use of antibacterial therapy among the two groups (p = 0.83), mechanical ventilation rates (p = 0.2), and length of hospital stay (p = 0.2) **(Table 4)**. The positive predictive value of PUAT was 69% in our study **(Table 5)**.

**Figure:**
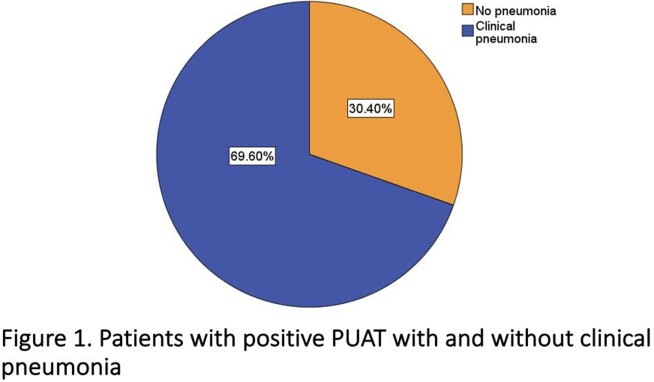

**Figure:**
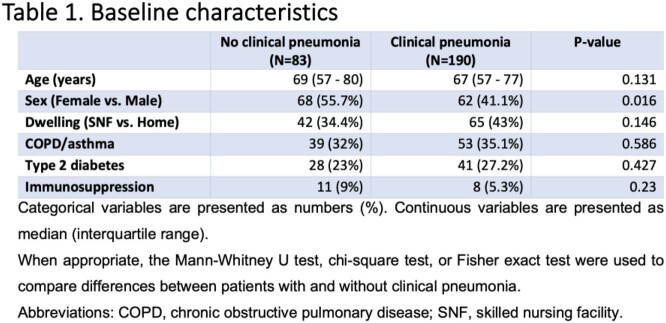

**Figure:**
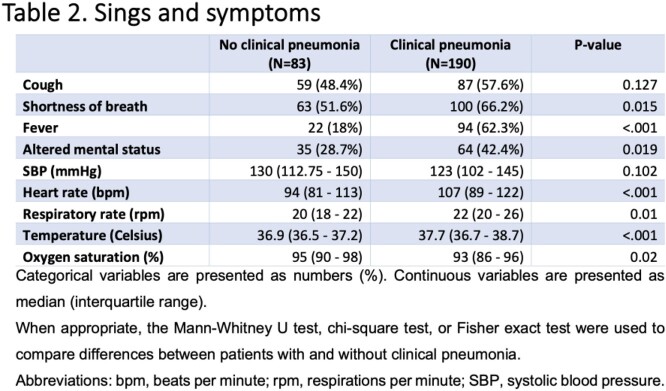

**Conclusion:**

Our study demonstrated a low positivity rate of PUAT despite a large sample size and a significant proportion of positive test results in patients without clinical pneumonia. The widespread use of PUAT should be carefully reconsidered and applied only to a selected category of patients. Such a strategy will reduce the discrepancy between current guidelines and real-world care patterns and provide an additional benefit of cost-effectiveness.

**Figure:**
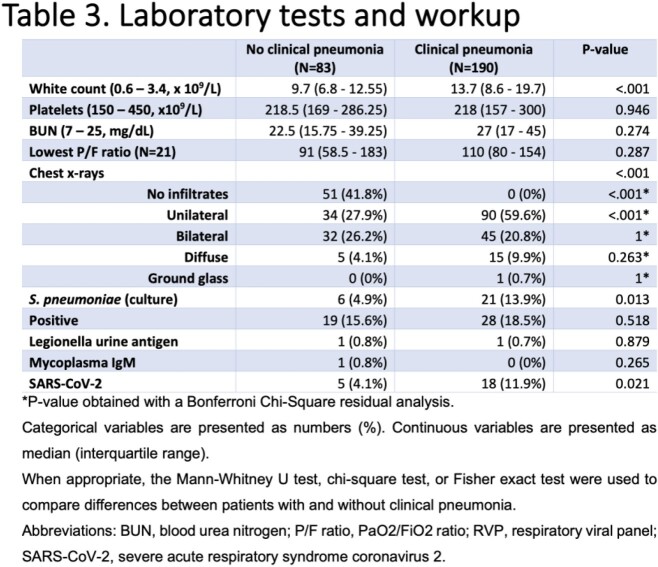

**Figure:**
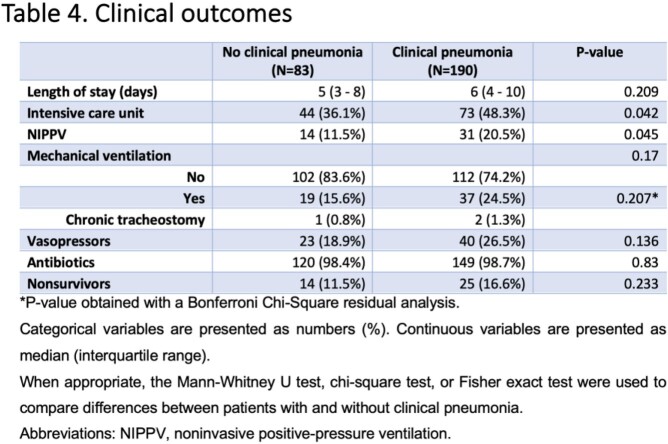

**Figure:**
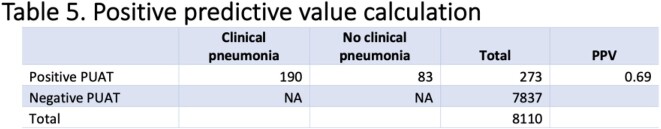

**Disclosures:**

**All Authors**: No reported disclosures.

